# Uganda’s cholera elimination journey in a cholera endemic region of Africa

**DOI:** 10.1371/journal.pgph.0006020

**Published:** 2026-05-21

**Authors:** Godfrey Bwire, David A. Sack, Allan Muruta, Charles Olaro, Jane Ruth Aceng Ocero, Diana Atwine, Henry G. Mwebesa, Francis Ongole, Bonny Kintu, Anne Nakinsige, Ampaire Immaculate, Buyinza Ambrose Wabwire, Rhoda K. Wanyenze, Amanda K. Debes, Christopher Garimoi Orach

**Affiliations:** 1 Department of Integrated Epidemiology, Surveillance Public Health Emergencies, Ministry of Health Uganda, Kampala, Uganda; 2 Department of International Health, Johns Hopkins Bloomberg School of Public Health, Baltimore, Maryland, United States of America; 3 Department of Finance and Administration, Ministry of Health, Kampala, Uganda; 4 Department of National Health Laboratory and Diagnostic Services, Ministry of Health, Kampala, Uganda; 5 Department of Urban and Regional Planning, Makerere University, Kampala, Uganda; 6 Department of Community Health and Behavioral Sciences, Makerere University School of Public Health, Kampala, Uganda; University of Health and Allied Sciences, GHANA

## Abstract

Uganda has been endemic for cholera since 1971. To reduce the threat of cholera, Uganda reviewed its approach to cholera, emphasizing surveillance, continued using single dose doxycycline for cholera patients and their households, and conducted 16 oral cholera vaccine campaigns in high-risk districts between 2018 and 2021.To understand recent changes in cholera patternsand the impact of the OCV campaigns, we classified districts as endemic, eliminated, or relapsed using the method as defined by the “cholera elimination scorecard” for the years 2018–2024 using cholera surveillance data from the Ministry of Health. We also reviewed Uganda’s policy regarding cholera surveillance and antibiotic use for cholera patients and their immediate households. To understand risks from cross-border cholera transmission, we reviewed cholera reports from the other countries in the East African Community. During the 16 campaigns with OCV (each campaign with two rounds), 2,263,790 people living in high risk subcounties of the hotspot districts were targeted for vaccination. Overall administrative vaccine coverage was 94% for the first round and 84% for the second round. Based on national surveillance data, the number of endemic districts fell from 36 prior to the campaigns to six after the campaigns. To identify new outbreaks and reduce the threat from cholera, Uganda implemented enhanced cholera surveillance, especially in the border areas, and administered single dose doxycycline to all cholera patients and their households with the intention to treat them and prevent spread of the outbreak. Since 2021, all cholera outbreaks in Uganda have been epidemiologically linked with cross-border spread from neighboring countries.Uganda has eliminated endogenous cholera (that is, cholera originating from within the country), but it remains vulnerable to cholera from neighboring countries in the region. Uganda’s experience to control cholera may be useful for other countries in Africa which are attempting to eliminate this disease.

## Introduction

Despite advances and innovations in public health, including efforts toward improved water, sanitation, and hygiene (WASH), and an efficacious oral vaccine and therapeutics, cholera remains a major threat, leading to high morbidity and mortality in many low-income countries. According to the World Health Organization (WHO), from January through August 2025, a cumulative total of 462,890 cholera cases and 5,869 deaths were reported from 32 countries across five WHO regions, with the African Region recording the second highest numbers [1].

The 2017 Global Task Force on Cholera Control (GTFCC) published a global roadmap which aimed to eliminate cholera from at least 20 countries and to reduce cholera mortality by 90% by the year 2030. The roadmap outlined general strategies to accomplish this ambitious goal including a) early detection and quick response to contain outbreaks, b) a targeted approach to improve prevention, and c) coordination of human technical and financial resources [2]. Yet, as the deadline approaches, a disturbing trend has emerged: cholera outbreaks continue to surge in many countries, including Africa, threatening to undermine decades of progress. These continued outbreaks suggest that a renewed effort is needed to accomplish the goals set by the GTFCC, and that some of the lessons from Uganda might be considered by other countries.

Uganda is a landlocked country in eastern Africa with a land area of around 241,550.7 square kilometers and population 45,905,417 persons as of May 2024 and an annual growth rate of 2.9 percent [3]. Uganda is a member State of the East African Community (EAC), a regional grouping that includes Kenya, Tanzania, Rwanda, Democratic Republic of Congo (DRC), South Sudan, Burundi, and Somalia [4].

**Fig 1:**
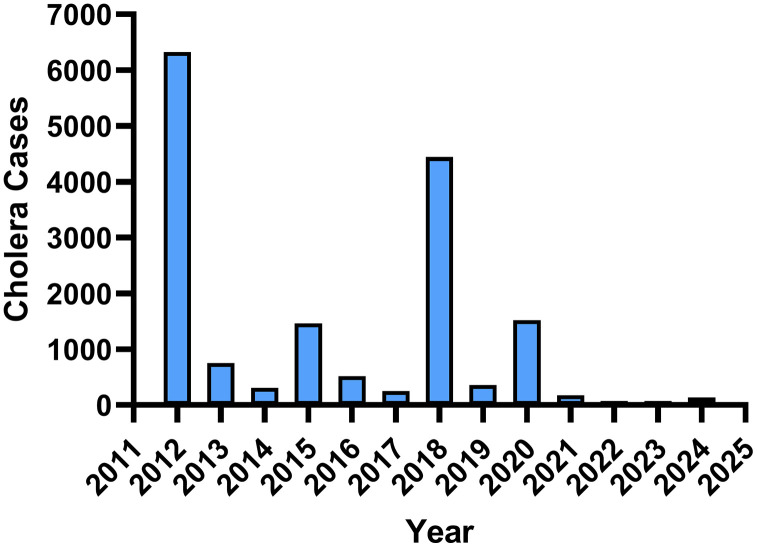
Number of Cholera Cases in Uganda 2012 -2024‌‌.

Uganda, like other countries in Sub-Saharan Africa has been endemic for cholera. The number of cholera cases between 2012 and 2024 is shown in Fig 1. Uganda is also surrounded by other cholera endemic countries in the East African Community and several of these countries are experiencing ongoing civil conflicts, resulting in their citizens becoming refugees migrating to other countries, like Uganda, that are willing to provide asylum [5]. Currently, Uganda hosts over 1.93 million refugees, from neighboring countries experiencing civil conflicts such as DRC, Burundi, South Sudan, Sudan, and others [6] and many of these countries have a large number of cholera cases [7].

In 2017, Uganda developed a National Integrated Comprehensive Cholera Prevention and Control Plan (NCP) [8]. The major cholera control actions in Uganda included a) social mobilization and community empowerment (health promotion & education for disease prevention); b) promotion of access to safe water, good sanitation and hygiene; c) surveillance and laboratory confirmation of outbreaks, d) prompt case management and infection control; e) complementary use of oral cholera vaccine (OCV) for cholera endemic communities; f) coordination and stewardship between and for all actors. g) monitoring, supervision, evaluation, and operation research to ensure continued improvement in service delivery.

Included in this strategy is the use of single dose doxycycline to treat cholera patients and their household members. As a follow-up to the 2017 national plan, in 2025, the leadership in the Ministry of Health emphasized its commitment by issuing detailed operational guidelines for national and district health workers for prevention, control and elimination of cholera [9].

**Fig 2:**
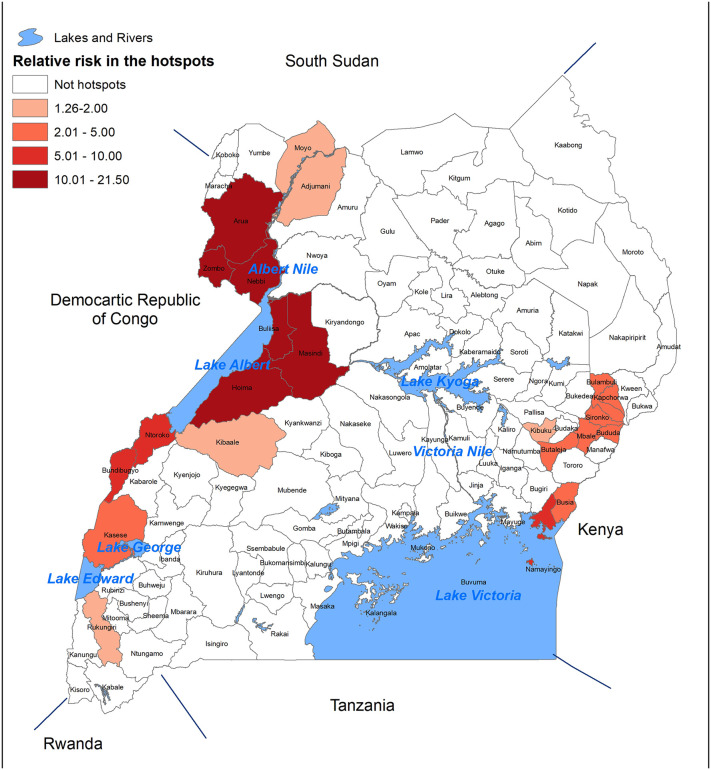
Cholera hotspot districts identified in Uganda between 2011 and 2016 using the SaTScan method [10].

Using the cholera surveillance data from the Ministry of Health the districts with higher cholera rates during the period from 2011 to 2016 were identified using SaTScan as shown in Fig 2 [10]. Between 2015 and 2019, four of the “hotspot districts” experienced outbreaks during two of the years, three experience outbreaks during three of the years, and one experienced an outbreak during four of the years. During a national cholera workshop in January 2018, the cholera hotspot map was discussed with key stakeholders including senior members from the Ministry of Health and representatives from the hotspot districts. These representatives provided insight into the specific subcounties within the hotspot districts which had the highest rates and were the “true hotspots.” The hotspot map, along with this local knowledge informed Uganda’s application for a series of OCV campaigns, which were planned to be implemented over a 3-to-4-year period targeting these specific subcounties in the hotspot districts.

Following the OCV campaigns, the numbers of cholera cases and the number of districts appeared to decline. To evaluate the changes in cholera patterns, we reviewed the cholera surveillance data from Uganda and the neighboring countries before and after the campaigns as well as the reports from the OCV campaigns.

Since many refugees enter Uganda each year, including some from cholera endemic areas, the procedures for managing their arrival should be noted. When they enter Uganda, the refugees report to a registration station and are screened for selected infectious and nutritional diseases. If diarrhea is reported, a stool sample is tested for cholera using a rapid diagnostic test (RDT, Crystal VC) with confirmation using an enriched RDT [11] and / or culture or PCR. In addition, hospitals, especially those close to the border, are on alert for cases of severe diarrhea and are able to test stool samples of patients with RDTs. If a single cholera case is confirmed, the Ministry of Health immediately responds to provide clinical care and additional resources for treatment and surveillance in the area. All isolates of *V. cholerae* are tested for antibiotic sensitivity including tests for tetracycline resistance.

Also to be noted, according to Uganda policy, patients with symptoms of cholera and with a positive RDT are treated with the appropriate rehydration fluids and are given a single dose of doxycycline. Because of the high risk that others in the household may also be infected with *V. cholerae*, [12] the other household members are also treated with a single dose of doxycycline to reduce the risk of onward spread. While using these procedures, there have been no reports of *V. cholerae* doxycycline resistant isolates in Uganda.

With the changing patterns of cholera in Uganda, we felt that a description of these changes following the implementation of OCV and control efforts was appropriate.

## Methods

### Uganda cholera surveillance

To determine the occurrence of cholera cases and trends over time, cholera surveillance data was extracted from the Uganda Ministry of Health surveillance reports for the period 2012 to mid-2025. For this surveillance, the case definition for a suspected case, in the absence of an outbreak, is a patient ≥5 years of age with severe watery diarrhea resulting in severe dehydration or death. During an outbreak the age is changed to ≥2 years. A confirmed case is a diarrhea patient with a positive stool culture for *V. cholerae* O1 or O139. An outbreak is declared when a cluster of cases occurs associated with a positive stool culture. The routine surveillance system thus reports diarrhea cases meeting the case definition that occur during outbreaks or from sporadic cases with a positive culture. Data were extracted for the following variables: number of reported cholera cases, the year when the outbreaks occurred, the location of the districts where cholera occurred, the dates and districts where OCV campaign were implemented and the type of campaign (reactive or preventive campaign).

### Cholera elimination scorecard

To monitor the annual changes in cholera patterns between 2018 and 2024, we used the scorecard method as previously described [13]. Briefly, using this method, we evaluated each district each year to determine if cholera is considered to be endemic in that district, or if cholera has been eliminated in the district. According to this method, a district is deemed to have **e*liminated*** cholera if it reports *no cases for four consecutive years inclusive of that year*. If cases are reported within the four-year period, the district is considered **endemic**. If after a district had eliminated cholera, cholera cases again are detected, the district is said to have ***relapsed***. By mapping changes in status of each district, the scorecard can be used annually to monitor progress toward national elimination. While the GTFCC determines a country to have eliminated cholera if there are no cases reported for three years, the score card method used four years as the time period in an attempt to set a higher standard when referring to a district rather than a country. We also compared the scorecard method to an alternative method recommended by the GTFCC which is used to identify hotspots, (now termed PAMIs (Priority Areas for Multisectoral Intervention)) [14]. (A similar method using SaTScan was used earlier when identifying the hotspots) [10]. The SaTScan and PAMI methods use data over multiple years to identify the districts which tend to have the highest rates and the most persistence over these years to identify those districts to focus interventions, including vaccine. To better understand the scorecard and the PAMI methods, we reviewed these methods with regard to the following attributes, a) frequency of assessment, b) ability to track progress toward the 2030 goals, c) ease of use, d) data requirements and e) the purpose for each method.

### Comparison of two reactive OCV campaigns

We also focused a specific review on the effectiveness of two OCV reactive campaigns conducted in Uganda during 2018–2019. These campaigns in Hoima and Bududa districts were selected because of their temporal proximity, and neither campaign was confounded by the impact of the COVID-19 pandemic which disrupted transportation and public health interventions globally including the East African region [15]. These two campaigns are described to illustrate issues regarding the time of vaccination when delivered in response to an outbreak.

### Cholera in the East African community

To understand the cholera burden in the other members of the East African Community, we reviewed and mapped the relevant data from the WHO cholera dashboard for the period from January 2023 to June 2025 [7].

### Data analysis

Data were collected, cleaned and stored in Microsoft Excel. Data were analyzed and presented in tables, graphs, and maps. Using data from WHO [7], the maps were generated using Arcgis Arcmap 10.8 software, version 10.8. shapefiles used to generate the maps were obtained from ICPAC Geoportal for EAC map and from UBOS for the map of Uganda (https://geoportal.icpac.net/ and https://www.ubos.org/data-portals-2/).

## Results

### OCV Campaigns in Uganda, 2018–2021

The National Cholera Control Plan (NCP), developed in 2017, defined strategies including surveillance, water-sanitation-hygiene, case management, risk communication, and community engagement but also included use of OCV as a complementary intervention. Following the approval of the OCV application in 2018, 12 preventive and four reactive campaigns were carried out between 2018 and 2021 including seven campaigns in the western region, five in the northern region, and four in the eastern region. Two rounds of vaccination were provided for each campaign. A total of 2.26 million people were targeted for vaccination, and the coverage rate overall was 94% for the first round and 84% for the second round. The first-round coverage rate ranged from 70% to 113% and the second-round coverage ranged from 69% to 118%. Details for each of the campaigns are found in Supplement Table 1.

### Changes in cholera patterns before and after the OCV campaigns

Using the cholera elimination scorecard, 36 districts out of 146 districts in Uganda were deemed to be endemic in 2018, the others were deemed to have eliminated cholera. This did not change in 2019, but in subsequent years, the number of endemic districts decreased progressively and there were just six districts deemed to be endemic in 2024 as shown in Table 1. This reduction was especially notable for the western districts near DRC that had repeatedly experienced outbreaks, but these districts have not detected cholera cases following the OCV campaigns.

**Table 1:** Number of endemic districts in Uganda using the scorecard method.

Year	2018	2019	2020	2021	2022	2023	2024
Number of districts with elimination	36	36	20	18	10	8	6
Number of relapsed districts		2	2	0	0	2	3

The total number of endemic districts decreased between 2018 and 2024. However, three of the six endemic districts identified in 2024 were districts that had relapsed since these were districts that experienced a small outbreak after having eliminated it. In fact, since 2021, all cholera outbreaks have been epidemiologically linked to cross-border spread from neighboring countries, especially with, but not exclusively with refugees; one outbreak in southern Uganda was traced to fishermen who work between Tanzania and Uganda [16].‌‌

### Two reactive campaigns

**Fig 3:**
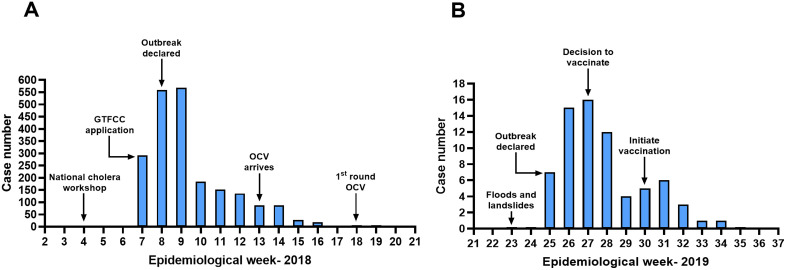
Example of the epicurves associated with two reactive oral cholera vaccine campaigns. (A) refers to Hoima, (B) refers to Bududa.

The epicurves and the course of the OCV campaigns for two of the reactive campaigns are shown in  Fig 3. The Hoima campaign was carried out in response to an outbreak that occurred in early 2018, shortly after the preventive OCV application was submitted but before it was approved. This outbreak led to an emergency application to the ICG (Interagency Coordinating Group) for reactive use of OCV, but unfortunately, in this case, the campaign could not be implemented until the outbreak was over. Even though the outbreak was over, the campaign continued as planned since this area had earlier been identified as a hotspot in the OCV application. In contrast to the campaign in Hoima, the reactive campaign in Bududa used doses of vaccine that were available in country because they were left over from a previous campaign. In effect, these doses constituted a type of small national stockpile and there was no delay in obtaining vaccine. The OCV campaign could be carried out quickly, and it appeared to have a benefit in shortening the course of the outbreak.

### Cholera in the East African community

**Fig 4:**
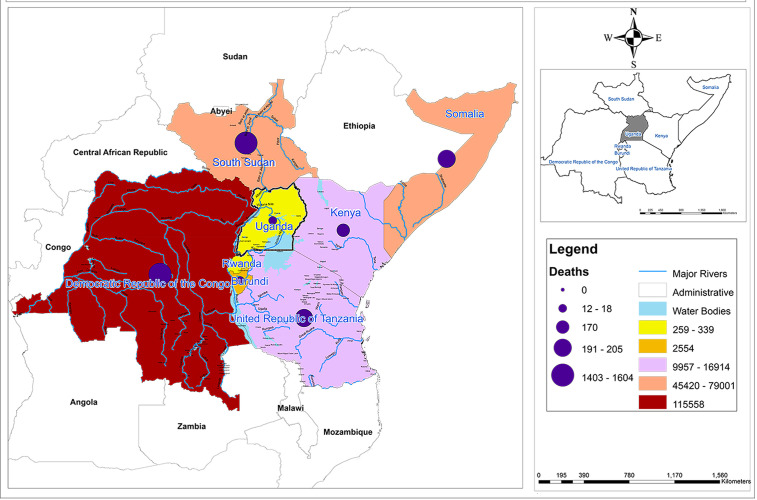
Map showing cholera risk of East African Community from January 2023 to June 2025 using data from the WHO Dashboard [7] and a shapefile from IGAD https://igad.int/.

While Uganda has experienced a marked reduction in the number of cases and the number of districts with cholera, nearly all of the other countries in the region (with the exception of Rwanda) continued to report large numbers of cases. Using data from the WHO cholera dashboard [7], cholera rates in the East African Community from January 2023 to June 2025 are shown in Fig 4.

### Scorecard and PAMI methods

A comparison of the PAMI and Scorecard methods is shown in Table 2. The PAMI method identifies areas of elevated risk as determined over multiple years and is used to guide increased resources, including vaccine, to the areas found to have the highest burden. Thus, it requires multiple year case data by month or week for each district. Preparing a national PAMI report requires considerable effort and training. However, since the PAMI method looks at trends over multiple years, it is not sensitive to rapid changes in the patterns that might result from effective interventions like a vaccine campaign.

**Table 2:** Comparison of scorecard and PAMIs for monitoring cholera elimination progress.

Item	Scorecard	PAMIs
District-level multi-year cholera surveillance data required	Yes, but only to determine the presence or absence of cholera in each district	Yes, average annual rates and numbers of cases monthly in each district
Frequency of assessment	Annual analysis to understand changes in cholera patterns based on multi-year data	5-year data is analyzed to understand which districts tend to have a higher burden
Useful to monitor annual changes	Yes	No
Ease of use	Analysis is relatively easy	Requires more training and analysis

The scorecard method also uses data over multiple years, but it shows the changes that occur from year-to-year rather than providing an overall (average) trend. Like the PAMI, it also requires district level data but only records data to show if cholera occurred in the district each year, but does not require a precise number of cases, so it is relatively easy to create maps illustrating where cholera is occurring, where it has been eliminated, and which districts relapsed after eliminating it.

## Discussion

Uganda has made considerable progress toward cholera elimination and based on national surveillance data, appears to have eliminated endogenous cholera. Endogenous cholera here is defined as the occurrence of cholera that originates from within its national borders. As of June 2025, our analysis indicates that no district in Uganda met the WHO criteria for cholera endemicity which is defined as “an area where confirmed cholera cases, resulting from local transmission, have been detected in the last 3 years. An area may be any subnational administrative unit including state, district or smaller localities. Any country having one or more subnational administrative units that are endemic, as defined above, is considered a cholera-endemic country” [17]. By contrast, other African countries, such as the DRC, reports many cases of cholera each year, and these cases appear to result from the continuous transmission of *V. cholerae* within its borders (though an environmental reservoir(s) have not been ruled out). In contrast to the DRC, all cholera cases in Uganda in recent years are epidemiologically linked to cross-border spread from people entering from a neighboring country. Some of these cases have resulted in localized outbreaks with transmission to the Ugandan population, but they have been contained within a small radius, generally within the same district.

The reasons for Uganda’s success are likely multi-factorial, including the high priority given to cholera control by the Ministry of Health, but the OCV campaigns targeted to the hotspot subcounties appear to have played a role since the districts in Western Uganda that had experienced repeated outbreaks have not experienced an outbreak since the campaigns. While the OCV campaigns may have provided protection for a few years, vaccine protection persists for only about 3–5 years [18,19], and the vaccine delivered in 2018–21 is unlikely to provide ongoing immune protection to those vaccinated earlier. Furthermore, population movement would have reduced the proportion of immunized individuals in these hotspot districts. Thus, these districts are again vulnerable to outbreaks should *V. cholerae*, once again be introduced into the area.

Most of the OCV campaigns in Uganda were preventive campaigns with only four campaigns being reactive, intended to control an outbreak. The reactive campaign in Hoima in 2018 could be initiated only after the last case in the outbreak occurred. Even though the outbreak was over, the vaccine was available, so it was used to vaccinate the hotspot subcounties in this district that had already been targeted for vaccine in the OCV application. In effect, this “reactive” campaign became a “preventive” campaign intended to stop the next outbreak. This experience illustrates the overlap between the two types of campaigns. Further, the experience of being able to use left over vaccine quickly for a new outbreak illustrates the potential benefit of a small national stockpile which can be deployed quickly.

To protect against new outbreaks, the Ministry has a robust surveillance system in which refugees are screened for diarrhea when they enter the country. Stools of diarrhea patients are tested using RDTs and RDT positive stool samples are confirmed positive using enriched RDTs, culture and since 2024, PCR. This enhanced surveillance allows for a rapid response by the Ministry to reduce the risk of deaths among the patients as well as reducing the risk for the outbreak spreading.

In Uganda, the treatment of cholera patients includes single dose doxycycline for both the cholera patient and members of the immediate household. Some have been concerned that this approach may lead to development of antibiotic resistance. In Uganda stool samples of all patients suspected of cholera are cultured and isolates are tested for antibiotic sensitivity. This focused single dose therapy, being limited to a single dose for the immediate household has not been associated with development of resistant strains of *V. cholerae*. Since household members are frequently infected with *V cholerae* and be a source for onward transmission [12], it is hoped that the single dose doxycycline for household contacts will stop this spread and limit the risk of larger scale outbreaks.

Of note, even before the OCV campaigns, most districts in Uganda were deemed to have eliminated cholera. The scorecard map of 2018 showed that only 36 of the 134 districts had endemic cholera. This made it possible to concentrate OCV to the few districts which repeatedly experienced cholera outbreaks.

While improvements in WASH are stressed as the primary strategy for cholera elimination, there is no indication that the districts that did experience cholera have WASH indicators that are different from the districts that did not experience cholera. This suggests that, although WASH improvements are needed for a host of reasons, including reducing the risk of future cholera outbreaks, these improvements will take time and resources, but controlling cholera can proceed while these WASH improvements are undertaken.

When comparing the scorecard and PAMI methods, these methods serve different purposes and the usefulness of each may differ depending on the country. The scorecard analysis is based on the presence or absence of cholera in each district each year and can show the changes from year to year, and especially to highlight relapsed districts which can then lead to investigations to understand the reason for the relapse. By contrast, the PAMI method would appear to be more useful for countries with a higher, more widespread, cholera burden (e.g., DRC) and identifies the districts that tend to have more cases where additional interventions, e.g., vaccine, are needed. In Uganda, to minimize the total number of OCV doses required, it was important to identify the smaller geographic units (the subcounties) as the true hotspots, rather than the entire district. Immunizing the entire districts would have needed many more doses of vaccine with a much greater requirement for additional national resources.

We feel this experience in Uganda which appears to have achieved endogenous elimination may provide lessons for other countries that are working toward elimination; however, the applicability depends on national context and surveillance capacity. These lessons include the targeting of OCV to the specific high risk areas within a hotspot district, the systematic use of OCV to vaccinate these areas over a multi-year concerted program, the benefits of a small national stockpile, an enhanced surveillance system utilizing RDTs to quickly identify and respond to an outbreak, and the use of single dose doxycycline for all cholera patients and their households to limit spread of the outbreak.

## Supporting information

S1 Table Districts in Uganda that received oral cholera vaccine (OCV) and the administrative vaccine coverage of each round. (DOCX)
