# Supplementary material for: Uganda’s cholera elimination journey in a cholera endemic region of Africa
Source: PLOS Glob Public Health. 2026 May 21;6(5):e0006020. doi: 10.1371/journal.pgph.0006020 (PMC13193533; doi:10.1371/journal.pgph.0006020)
Supplement: S1 Table — (DOCX) [file pgph.0006020.s001.docx]

| **District** | **Region** | **Population** | **Type of campaign** | **Dates (dose 1)** | **Dates (dose 2)** | **Target Population** | **Coverage dose 1** | **Coverage dose 2** |
| --- | --- | --- | --- | --- | --- | --- | --- | --- |
| Hoima/Kikuube | Western | Ugandan citizen and refugees | Reactive | May 2018 | Jun 2018 | 383,228 | 93% | 85% |
| Buliisa (3 subcounties) | Northern | Ugandan citizen | Preventive | Apr 2019 | Dec 2019 | 128,103 | 96% | 80% |
| Buliisa (4 subcounties) | Western | Ugandan citizen | Preventive | Dec 2019 | Feb 2020 |  |  |  |
| Nebbi | Northen | Ugandan citizen | Preventive | Apr 2019 | Dec 2019 | 179,663 | 113% | 69% |
| Pakwach | Northen | Ugandan citizen | Preventive | Apr 2019 | Dec 2019 | 170,846 | 107% | 97% |
| Zombo | Northen | Ugandan citizen | Preventive | May 2019 | Dec 2019 | 151,508 | 84% | 118% |
| Bududa | Eastern | Ugandan citizen | Reactive | Jul 2019 | Dec 2019 | 51,825 | 113% | 94% |
| Moroto | Northern | Ugandan citizen | Reactive | Jun 2019 | Sep 2020 | 94,954 | 70% | 87% |
| Busia | Eastern | Ugandan citizen | Preventive | Oct 2020 | Sep 2021 | 71,743 | 85% | 86% |
| Namayingo | Eastern | Ugandan citizen | Preventive | Oct 2020 | Sep 2021 | 157,249 | 98% | 86% |
| Obongi | Northern | Ugandan citizen | Preventive | Oct 2020 | Sep 2021 | 144,222 | 86% | 11% |
| Madi-Okollo | Northern | Ugandan citizen | Preventive | Oct 2020 | Sep 2021 | 196,663 | 99% | 99% |
| Ntoroko | Western | Ugandan citizen | Preventive | Oct 2020 | Sep 2021 | 72,576 | 97% | 93% |
| Kasese | Western | Ugandan citizen | Preventive | Oct 2020 | Sep 2021 | 396,000 | 90% | 87% |
| Isingiro | Western | Refugees | Reactive | Nov 2021 | Dec 2021 | 14,335 | 75% | 85% |
| Isingiro | Western | Refugees | Preventive | Sep 2022 | Sep 2022 | 49,875 | 100% | 81% |
| **Total target population and average vaccine coverage** | | |  |  |  | **2,262,790** | **94%** | **84%** |
